# Proteomic analysis of decidua in patients with recurrent pregnancy loss (RPL) reveals mitochondrial oxidative stress dysfunction

**DOI:** 10.1186/s12014-021-09312-2

**Published:** 2021-02-22

**Authors:** Xiang-Jie Yin, Wei Hong, Fu-Ju Tian, Xiao-Cui Li

**Affiliations:** 1 Department of Gynecology, Shanghai First Maternity and Infant Hospital, Tongji University School of Medicine, Shanghai, China; 2 International Peace Maternity and Child Health Hospital, School of Medicine, Shanghai Jiao Tong University, Shanghai, China

**Keywords:** Proteomics, Decidua, ndufb3, NDUFB3, Recurrent pregnancy loss (RPL)

## Abstract

**Background:**

Pregnancy is a complicated physiological process. The multifaceted regulation of maternal–fetal interface is of great importance for maintaining normal pregnancy and avoiding fetal rejection and secondary abortion. Previous studies have focused on the clinical features or pathological biomarkers of fetal rejection and abortion. However, no significant breakthrough has been made. Therefore, it is important to understand the molecular mechanisms of recurrent pregnancy loss (RPL) to identify potential therapeutic strategies. The aim of this study was to investigate the pathogenesis of RPL.

**Methods:**

In this study, Relative and absolute quantitation (iTRAQ) technology integrated with liquid chromatography-tandem mass spectrometry (LC–MS/MS) analysis was used to identify differentially expressed proteins in decidual from RPL patients and matched normal controls. Further, Molecules NADH dehydrogenase [ubiquinone] 1 beta subcomplex subunit 3 (ndufb3) and cyclooxygenase-2 (COX-2) were validated by immunohistochemistry (IHC), Western blotting, CCK8 and mitochondrial red fluorescent probe (Mito-Tracker Red CMXRos).

**Results:**

A total of 456 proteins reached the threshold of a 1.5-fold change were identified for further bioinformatics analysis. Upon mapping the differentially expressed proteins using the Kyoto Encyclopedia of Genes and Genomes (KEGG) pathways database, iTRAQ results were confirmed by assessing NDUFB3 and COX-2 protein levels in specimens of decidual tissue by Western blotting. Our study indicates that the level of COX-2 and NDUFB3 were significantly increased in decidual cell from RPL patients. Overexpression of NDUFB3 inhibited cell vitality and oxidative stress of decimal cell. Further, our found that overexpression NDUFBD3 in decidual cell decreased the mitochondrial membrane potential expression levels. These results suggest that NDUFB3 might play an important role in promote the pathological process of RPL.

**Conclusions:**

This comprehensive analysis of RPL proteomics reveals novel candidate: NDUFB3, which could be further investigated for explanation of the pathological mechanism of RPL.

## Background

Recurrent pregnancy loss (RPL) is defined as two or more consecutive spontaneous abortions before 20 weeks of gestation [1]. RPL affects approximately 1–3% of couples trying to conceive and is becoming both a clinical problem and a psychological stressor for the couples involved [2]. However, half of all patients with RPL do not know the underlying risk factors of their recurrent miscarriages [3]. Unexplained RPL is typically diagnosed after excluding other risk factors such as genetic, immunological, anatomical, endocrine, or placental anomalies; hormonal problems; infection; smoking and alcohol consumption; environmental factors; psychological trauma; and stressful life events [4–6]. Given the complexities of early development, it is likely that many mechanisms are involved in the pathophysiology of RPL. Therefore, further exploration of the etiology of RPL is critical.

A successful pregnancy involves a complex molecular dialogue between the maternal endometrium (decidua), the conceptus and the placenta. During embryonic implantation and placental formation, the endometrium undergoes extensive cyclic biochemical and morphological modifications. This process is referred to as the decidualization of stromal cells and is critical for the embryo to adhere to and invade the uterine epithelium [7, 8]. The decidua, which secretes products (e.g., fibronectin and insulin-like growth factor binding protein-1 [IGFBP1]) that bind to trophoblast-specific integrins and modulate trophoblast migration and invasion, is vital for supporting embryonic growth and maintaining early pregnancy [9, 10]. A growing number of reports attribute the dysfunction of human decidua to a variety of reproductive diseases and pregnancy complications, such as RPL [11, 12].

Proteomics, the large-scale study of proteins, contributes greatly to our understanding of gene function in the postgenomic era. Tandem mass tags (TMTs) are an excellent tool for proteomic analysis and can be used to compare patterns of protein expression in cells under various physiological and pathological conditions [13]. Thus, proteomics, in conjunction with high-throughput polymorphism analysis, may enable us to unravel the specific molecular complexes or pathways that are involved in the pathogenesis of RPL.

In this study, we analyzed decidua from normal early pregnancies and recurrent pregnancy loss. TMT sequencing was used to identify and analyze proteins and to establish differential proteomic profiles for decidua from normal and aborted pregnancies. We also examined the functions of these differentially expressed proteins in the maintenance of early pregnancy and the occurrence of RPL.

Mass spectrometry (MS)-based proteomics plays a key role in the identification of biomarkers useful for the diagnosis and prognosis of many diseases, including recurrent spontaneous abortion. MS-based proteomics involves the separation of intact proteins or peptides, which are generated by trypsin treatment. This separation is carried out using various techniques, the most common of which are two-dimensional gel electrophoresis and multidimensional nanoflow HPLC. Finally, peptides are identified by tandem mass spectrometry. In the case of biomarkers, polypeptide [14, 15] expression levels must be quantified. Several quantification strategies have been used to identify RPL biomarkers, including isobaric tag for relative and absolute quantitation (iTRAQ).

As we all know, mitochondrial respiratory chain dysfunction is an important risk factor of various human diseases. Its prevalence is approximately 1/5000 in both adults and children. Symptoms may be evident during the neonatal period, however onset more often occurs during late infancy, early childhood and even adulthood. Patients with mitochondrial respiratory chain dysfunction may develop diseases that affect a single organ or may develop multisystem diseases such as Leigh syndrome [16]. Approximately 70% of cases of pediatric mitochondrial disease are due to nuclear gene variants, and approximately 30% of all genetic defects involve mitochondrial coding genes. Mitochondrial DNA mutations are more often the basis for manifestations of adult mitochondrial disease.

## Materials and methods

### Decidual tissue samples

The RPL group consisted of three patients who had experienced two or more consecutive pregnancy loss during the first trimester of pregnancy (age: 29.13 ± 3.31 years; gestational age: 57.25 ± 9.16 days). Participants in the RPL group were characterized as having had an abortion of unexplained etiology, a history of miscarriage, and no chromosomal abnormalities. The control group consisted of three age-matched women with normal pregnancies who were undergoing artificial miscarriage (elective abortion) (age: 28.75 ± 2.43 years; gestational age: 58.5 ± 4.44 days) (Table 1). The inclusion criteria were as follows: gestational age of 6–13 weeks. Patients were excluded based on the following criteria: history of other gynecological diseases, internal diseases, maternal anatomical abnormality, and any chemical agent intake before termination. Participants did not smoke cigarettes or drink alcohol. Decidual tissues were collected, frozen in liquid nitrogen, and then immediately stored at − 80 °C until analysis. Informed consent was obtained from each woman. Approval was obtained from the Ethical Committee of Shanghai First Maternity and Infant Hospital, Tongji University School of Medicine.

**Table 1:** Characteristics of the RSA and Control group in the present study (x ± s)

	Normal (n = 6)	RPL (n = 6)	p value
Maternal age (year)	34.17 ± 3.60	34.67 ± 2.80	> 0.05
Height (cm)	158.58 ± 5.54	162.50 ± 4.72	> 0.05
Weight (kg)	54.17 ± 10.46	56.83 ± 7.00	> 0.05
BMI (kg/m^2^)	21.49 ± 3.73	21.54 ± 2.69	> 0.05
Gestational age (day)	75.67 ± 17.92	73.33 ± 13.29	> 0.05

### Proteomics experiments

#### Reduction, alkylation, digestion and TMTsixplex™ labeling

TMTsixplex™ experiments were carried out according to the manufacturer’s instructions. In this experiment, we analyzed samples from three RPL patients and three control patients (each group of samples were obtained from three individuals and was randomly divided into 3 biological replicates). SDT buffer was added to each sample. Lysates were sonicated (this step was skipped for protein solutions) and then boiled for 15 min. Samples were centrifuged at 14000*g* for 40 min, and then the protein concentration of the supernatant was quantified using the BCA Protein Assay Kit (Bio-Rad, USA). 200 μg of each protein sample was brought to 30 μL using SDT buffer (4% SDS, 100 mM DTT, 150 mM Tris–HCl pH 8.0). Detergent, DTT and other low-molecular-weight components were removed using UA buffer (8 M Urea, 150 mM Tris–HCl pH 8.0) and repeated ultrafiltration (Microcon units, 10 kD). Then, samples were incubated for 30 min in the dark with 100 μl iodoacetamide (100 mM IAA in UA buffer) to block reduced cysteine residues. The filters were washed three times with 100 μl UA buffer and then twice with 100 μl 100 mM TEAB buffer. Finally, the protein suspensions were digested with 4 μg trypsin (Promega) in 40 μl TEAB buffer overnight at 37 °C, and the resulting peptides were collected as a filtrate. Peptide content was estimated by UV light spectral density at 280 nm using an extinction coefficient of 1.1 in a 0.1% (g/l) solution (calculated based on the frequency of tryptophan and tyrosine residues in vertebrate proteins). 100 μg of each peptide mixture was labeled using the TMT reagent according to the manufacturer’s instructions (Thermo Fisher Scientific).

#### Peptide fractionation

The Pierce High pH Reversed-Phase Fractionation Kit (Thermo Scientific) was used to fractionate TMT-labeled digested samples into 15 fractions using an increasing acetonitrile step-gradient elution according to the manufacturer’s instructions.

#### Mass spectrometry

##### HPLC

Each fraction was injected for nanoLC-MS/MS analysis. The peptide mixture was loaded in buffer A (0.1% formic acid) onto a reversed-phase trap column (Thermo Scientific Acclaim PepMap100, 100 μm*2 cm, nanoViper C18) connected to a C18 reverse-phase analytical column (Thermo Scientific Easy Column, 10 cm long, 75 μm inner diameter, 3 μm resin). Peptides were separated using a linear gradient of buffer B (84% acetonitrile and 0.1% Formic acid) at a flow rate of 300 nl/min controlled by IntelliFlow technology. The 1 h linear gradient was performed as follows: 0–50% buffer B for 50 min, 50–100% buffer B for 5 min, hold in 100% buffer B for 5 min.

##### LC–MS/MS analysis

LC–MS/MS analysis was performed on a Q Exactive mass spectrometer (Thermo Scientific) coupled to the Easy nLC system (Proxeon Biosystems, now Thermo Fisher Scientific) for 60/90 min (determined by project proposal). The mass spectrometer was operated in positive ion mode. MS data was acquired using a data-dependent top10 method to dynamically choose the most abundant precursor ions from the survey scan (300–1800 m/z) for HCD fragmentation. The automatic gain control (AGC) target was set to 3e6, and the maximum injection time was set to 10 ms. The dynamic exclusion duration was 40.0 s. Survey scans were acquired at a resolution of 70,000 at m/z 200 and the resolution for HCD spectra was set to 17,500 at m/z 200 (TMT 6plex) with an isolation width of 2 m/z. Normalized collision energy was 30 eV and the underfill ratio, which specifies the minimum percentage of the target value likely to be reached at maximum fill time, was defined as 0.1%. The instrument was run with peptide recognition mode enabled.

#### Protein search

Raw files were run through the Mascot engine (Matrix Science, London, UK; version 2.2) against the UniProt_human_156639_20170105.fasta database embedded into Proteome Discoverer 1.4. Trypsin was selected as the enzyme and only two mis-cleavages were allowed. The precursor mass tolerance and the fragment mass tolerance were set to 20 ppm and 0.1 Da respectively. The fixed modifications were carbamidomethyl on cysteine residues (57.02 Da) and TMTsixplex™ tags on lysine residues and peptide N termini (229.16 Da), while the dynamic modification was oxidation on methionine residues (15.99 Da). Target False Discovery Rate (FDR) for PSMs was set to 0.01. We used only unique peptides with a high confidence value and a minimum length of 6 amino acids. For quantification, only peptides that are not shared between different proteins or protein groups were used. Proteins with missing values were not replaced and were therefore rejected for quantification. The abundances of individual peptides were normalized using the total peptide quantity and scaled abundances were used for ratio calculations.

#### Data availability statement

Raw mass spectrometry data are available on the Proteome Xchange Consortium via the PRIDE database (project accession number: PXD006871).

### Bioinformatic analysis

#### Gene ontology (GO) annotation

The protein sequences of differentially expressed proteins were retrieved in batches from the UniProtKB database (Release 2017_01) in FASTA format. The retrieved sequences were locally searched against the SwissProt mouse database using the NCBI BLAST + client software (ncbi-blast-2.2.28 + -win32.exe) to find homologous sequences from which functional annotations could be transferred to the studied sequences. In this work, the top 10 blast hits with E-values less than 1*e*-3 for each query sequence were retrieved and loaded into Blast2GO [17] (Version 3.3.5) for GO mapping and annotation. Our annotation configuration was as follows: E-value filter of 1*e*-6, default gradual EC weights, GO weight of 5, and an annotation cutoff of 75. Unannotated sequences were then reannotated with more permissive parameters. Sequences without BLAST hits and unannotated sequences were then run through an InterProScan [18] against EBI databases to retrieve functional annotations of protein motifs. InterProScan GO terms were then merged with the annotation set. The GO annotation results were plotted using R scripts.

#### KEGG pathway annotation

The FASTA protein sequences of differentially expressed proteins were blasted against the online Kyoto Encyclopedia of Genes and Genomes (KEGG) database (http://geneontology.org/) to retrieve their KOs and were subsequently mapped to pathways in KEGG [19]. The corresponding KEGG pathways were extracted.

#### Functional enrichment analysis

To further explore the impact of differentially expressed proteins on cell physiological process and to discover relationships between differentially expressed proteins, enrichment analysis was performed. GO enrichment in three ontologies (biological process, molecular function, and cellular component) and KEGG pathway enrichment analyses were applied based on Fisher exact test, considering the total quantified protein annotations as the background dataset. Derived p-values were further adjusted using the Benjamini–Hochberg correction for multiple testing. Functional categories and pathways with p-values < 0.05 were considered to be significant.

#### Hierarchical clustering

Relative protein expression data was used to perform hierarchical clustering analysis using Cluster 3.0 (http://bonsai.hgc.jp/~mdehoon/software/cluster/software.htm) and the Java Treeview software (http://jtreeview.sourceforge.net). A Euclidean distance algorithm was used for similarity measurements and an average linkage clustering algorithm (clustering uses the centroids of the observations) was used for clustering when performing hierarchical clustering analyses. Heatmaps are presented as visual aids in addition to the dendrograms.

#### Protein–protein interaction network (PPI)

Protein–protein interaction information for the studied proteins was retrieved from the IntAct molecular interaction database (http://www.ebi.ac.uk/intact/) using gene symbols. The results were downloaded in XGMML format and imported into Cytoscape software (http://www.cytoscape.org/,version 3.2.1) to visualize and further analyze functional protein–protein interaction networks. The degree of each protein was calculated to evaluate the importance of the protein in the PPI network.

### Primary culture of decidual stromal cells

Decidual stromal cells (DSCs) were isolated according to the methods described in Li et al. [20]. Decidual tissues from different subjects were carefully freed from the trophoblast and then fully washed in Ca2^+^Mg2^+^-free PBS with 100 U/ml penicillin and 50 μg/ml gentamicin. Tissues were minced and then digested in a solution of 0.25% trypsin and 0.025% EDTA for 10 min at 37 °C. The enzymatic reaction was stopped by adding cold DMEM medium with 20% fetal bovine serum. The digestion reaction was repeated three times. The suspension was passed through sterile filters (100 μm and 300 μm pore sizes) and then centrifuged at 400 g for 10 min. The supernatant was discarded, and the cell pellet was resuspended in PBS and centrifuged through a discontinuous gradient of 20%, 40% and 60% Percoll (P7828, Sigma Aldrich) for 20 min at 800 g. Cells (mainly DSCs) were collected from the 20% and 40% interfaces, resuspended in DMEM, washed, and cultured in complete DMEM medium with 10% fetal bovine serum (FBS, S1810, Biowest, Nuaillé, France). After 6 h in culture, non-adherent cells were removed, leaving a highly purified, leukocyte-free population of DSCs.

### Overexpression of NDUFB3 and construction of adenovirus

Recombinant adenovirus containing the NDUFB3 gene was purchased from Obio Company (Obio Technology, Shanghai, China). pAdeno-MCMV-NDUFB3-P2A-EGFP (Ad-NDUFB3) and control adenovirus (Ad-GFP) were constructed, packed into adenovirus, and purified. The Ad-NDUFB3 and Ad-GFP with a titer of 2 × 10^10^ PFU were constructed by Obio Technology Corp., Ltd., Shanghai, China. DSCs were infected with Ad-NDUFB3 or Ad-GFP and collected at 48 h after infection. All the experiments were repeated at least three times with 2–3 samples per group at each time.

### Adenoviral infection of primary decidual stromal cells

First, decidua cells with good growth status were obtained. Then, DSCs were seeded in a 6 cm dish at a density of 6–8 * 10^4^ in order to ensure that the cell density would be between 50–70% at the time of infection. Immediately prior to infection, the culture medium was replaced with serum-free culture medium. 2 µl/well adenovirus was added to the serum-free culture medium and plates were shaken every half hour to increase the virus infection efficiency. Finally, after 2 h, 1 ml normal medium containing 10% FBS was added to each well. Fluorescence expression was observed 48–72 h after adenovirus infection.

### Western blot analysis

Frozen human decidua was homogenized in RIPA lysis buffer combined with PMSF and protease inhibitors (RIPA: PMSF: protease inhibitors = 100:1:1). Samples were centrifuged at 12,000 rpm for 30 min at 4 °C and then the supernatants were solubilized. 40 µg of each protein sample was separated by SDS-PAGE and transferred onto PVDF membranes (Bio-Rad Laboratories Inc., Hercules, CA, USA). Membranes were blocked for 1 h with 5% BSA in PBS containing 0.05% Tween-20 (PBST). Membranes were then incubated overnight at 4 °C in a solution of primary antibodies (NDUFB3, 1:1000, COX2, 1:1000) in 5% milk. Membranes were washed three times and then protein intensities were measured and analyzed using ECL reagent (Thermo Fisher, Waltham, MA, USA).

### Immunohistochemical staining for NDUFB3

Paraffin-embedded human decidual tissue sections (5 mm) were dewaxed with xylene, rehydrated in a descending alcohol gradient (from 100% ethanol to phosphate-buffered saline), and quenched in 0.3% hydrogen peroxide in methanol for 20 min. Antigen retrieval was performed in 10 mmol/L citrate buffer in a microwave oven. Sections were blocked with donkey serum and then incubated overnight at 4  C in primary rabbit monoclonal anti-human NDUFB3 antibody (ab202585; Abcam) diluted 1:200. Next, the slides were washed in phosphate buffered saline with Tween 20 and incubated with secondary goat anti-rabbit antibody. Immunostaining was performed in triplicate. Rabbit IgG was substituted for primary antibody in the negative controls.

### Cell proliferation assay

After transfection about five thousand decidual cells per well were plated in 96-well plates. 10 μl per well the cell counting kit-8 (CCK-8) regents (Dojindo, Kumamoto, Japan) were added to cells to assess cell viability at 0, 24, 48 and 72 h. After incubation with CCK-8 reagent for 2 h, the optical density value was detected at 450 nm using the SpectraMax M5 (Molecular Devices, United States).

### Mitochondrial membrane potential

Mito-tracker Red CMXRos is a mitochondria-specific fluorescent probe, whose staining of mitochondria depends on mitochondrial membrane potential. Therefore, mito-tracker Red CMXRos was used as an indicator probe to detect apoptosis by detecting changes in mitochondrial membrane potential. Firstly, decidual cells (3–4*10^6^) were harvested and then seeded in a 96-well plate at a density of 1*10^5^ cells/well. Secondly, the mito-Tracker Red CMXRos fluorescent probe was placed at 100 µl per well for 48 h. Cells were stained with mito-Tracker Red CMXRos fluorescent probe for 30 min at 37 ℃. Thirdly, the medium was wiped off and the signal was read at an excitation wavelength was 575 nm and the emission wavelength was 590 nm. Finally, the mitochondria of living cells stained with Mito-Tracker Red CMXRos were photographed by Leica DMi8 microscope (Wetzlar, Germany). All explant experiments with cultured decidual cells were repeated three times.

### H_2_O_2_ assays

H_2_O_2_ was measured using a Hydrogen Peroxide Assay Kit (Beyotime Biotechnology) according to the manufacturer’s instructions. After treatment for 48 h, 5 × 10^5^ cells were collected and lysed to measure H_2_O_2_ levels in vitro.

### Statistical analysis

Differences are expressed as the mean ± SEM in all experiments. Statistical differences were determined by Student t-test for two-group comparisons or analysis of variance (one-way ANOVA) followed by Dunnett test for multiple comparisons among more than two groups. These procedures were undertaken using PRISM software version 5.0; GraphPad. Significance level was set at a p value of < 0.05.

## Results

### Global protein profiling in decidua

We first sought to reveal proteomic changes in human decidua following recurrent pregnancy loss (RPL). Isobaric tags for relative and absolute quantitation (iTRAQ) technology integrated with liquid chromatography-tandem mass spectrometry (LC–MS/MS) analysis was used to quantitatively detect and map proteins in the human decidua. Using this unbiased proteomic analysis, we identified 4524 polypeptides with high confidence values (one or more unique peptides with an FDR less than 1%).

### Identification of significantly differentially expressed proteins in decidua

**Fig. 1:**
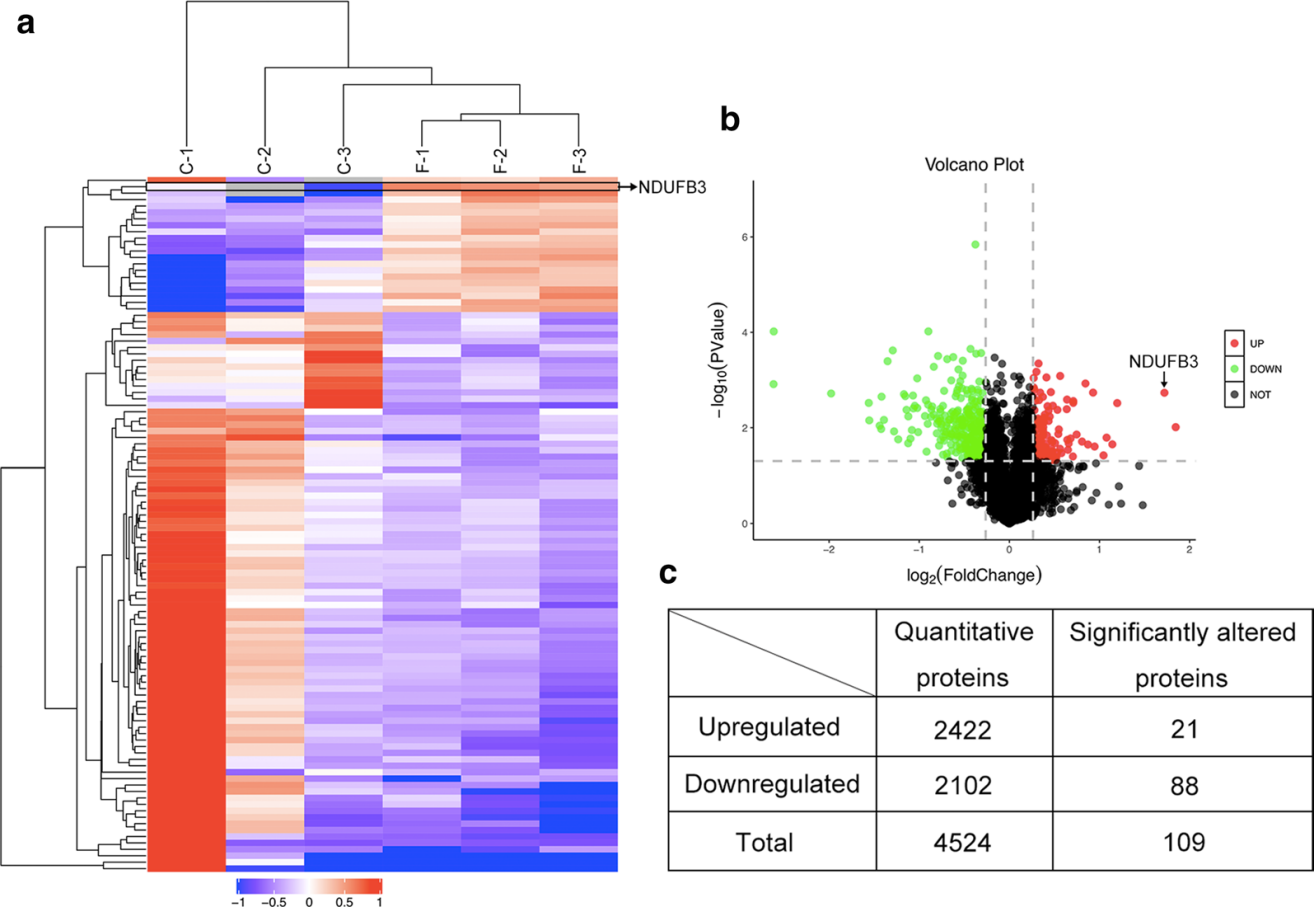
The decidual tissues of RPL were analyzed by proteomics. Samples of deciduas from 6–13 week RPL and normal pregnant women were irrespectively collected and comparatively analyzed by proteins sequencing. **a** Heatmap of normalized expression levels of decidual cell genes isolated from RPL patients (F-1, F-2, F-3) and control patients (C-1, C-2, C-3). Blue indicates low expression levels; red indicates high expression levels. **b** Volcano plot of the expression of altered proteins. **c** The expression of significantly up-regulated and down-regulated proteins

There are few reports of the application of quantitative proteomics to RPL tissue using iTRAQ and tandem mass spectrometry [21–23]. In this paper, we determined the protein expression profiles of RPL tissue. Specifically, we identified 4524 polypeptides labeled with the isobaric tags with at least one identified peptide with a confidence level equal to or higher than 95%. Of these, 456 proteins displayed expression level changes with high confidence values (one or more unique peptides with an FDR less than 1%). After further characterizing the specific and unique expression patterns of the 456 proteins, we were able to group these proteins into two clusters according to their expression patterns (upregulated and downregulated expression patterns). 133 proteins displayed the upregulated expression pattern, while 323 proteins displayed the downregulated expression pattern. The K-means clustering of these differentially expressed proteins can be visualized using a heatmap (Fig. 1a). The volcano plot displays the accumulation patterns of differentially regulated proteins in decidua (Fig. 1b). Based on WEKA software analysis, we selected 109 significantly differentially expressed proteins. These significantly up/downregulated proteins are shown in Table 2. Of this list of differentially expressed proteins, we propose that NDUFB3 may be a putative biomarker for RPL.

**Table 2:** Differentially expressed proteins in deciduas identified from TMT analysis

Accession	Description	Fold change	p value
Q9ULF5	Zinc transporter ZIP10	3.593601451	0.00974423
C9JKQ2	NADH dehydrogenase [ubiquinone] 1 beta subcomplex subunit 3 (Fragment)	3.295099345	0.00184999
Q6N091	Putative uncharacterized protein DKFZp686C02220 (Fragment)	2.292482498	0.00304797
B4DET8	cDNA FLJ57956	2.207706072	0.0223015
P02768	Serum albumin	2.112053053	0.016162
H0YED9	Wilms tumor protein (Fragment)	2.06273219	0.0380685
Q6N089	Uncharacterized protein	1.925515551	0.024817
P01859	Ig gamma-2 chain C region	1.903435322	0.00183541
B4DN39	cDNA FLJ53065, highly similar to T-complex protein 1 subunit zeta	1.830548838	0.0225267
Q8N355	IGL@ protein	1.795266303	0.00118425
H0YKW9	Glycine amidinotransferase, mitochondrial	1.748535612	0.0195367
F6KPG5	Albumin (Fragment)	1.677222309	0.0139091
Q8N692	Putative zinc finger protein (Fragment)	1.635176184	0.00276574
A0A0K0K1H8	Epididymis secretory sperm binding protein Li 71p	1.634622043	0.0030154
Q86Y01	E3 ubiquitin-protein ligase DTX1	1.63001989	0.0400716
Q6K0P9	Pyrin and HIN domain-containing protein 1	1.601769684	0.0167988
Q9BU23	Lipase maturation factor 2	1.577983128	0.0213518
B7ZBB8	Protein phosphatase 1 regulatory subunit 3G	1.569705967	0.029167
P67775	Serine/threonine-protein phosphatase 2A catalytic subunit alpha isoform	1.561790335	0.0179526
U3KQN5	Ribosome production factor 2 homolog	1.558050675	0.00257587
Q9BZK3	Putative nascent polypeptide-associated complex subunit alpha-like protein	1.554627182	0.00408793
P15153	Ras-related C3 botulinum toxin substrate 2	0.665577355	0.0240501
B7Z570	cDNA FLJ53078, highly similar to Splicing factor, arginine/serine-rich 1	0.66556235	0.00884293
B4E1B3	cDNA FLJ53950, highly similar to Angiotensinogen	0.664222282	0.0332029
P35908	Keratin, type II cytoskeletal 2 epidermal	0.661835288	0.00220015
I3L1H3	Lipopolysaccharide-induced tumor necrosis factor-alpha factor	0.661011033	0.0176498
A0A024R8I2	Ubiquitin associated domain containing 1, isoform CRA_c	0.661004813	0.0117604
H3BN55	Ras-related protein Rab-27A (Fragment)	0.660190749	0.00500513
Q6ZRP7	Sulfhydryl oxidase 2	0.65821681	0.0104548
Q5JZ02	TP53-regulating kinase	0.654150741	0.00639221
Q15080	Neutrophil cytosol factor 4	0.652148672	0.00712164
Q7RTS7	Keratin, type II cytoskeletal 74	0.65199753	0.00559901
U3PXP0	Alpha globin chain (Fragment)	0.649463918	0.000330814
H6VRF8	Keratin 1	0.647386165	0.00461357
Q9UPN1	Serine/threonine-protein phosphatase (Fragment)	0.642306447	0.0137497
B7Z8T3	cDNA FLJ50352, highly similar to Fetuin-B	0.640612662	0.0305719
Q9Y285	Phenylalanine–tRNA ligase alpha subunit	0.639955836	0.00101929
P14598	Neutrophil cytosol factor 1	0.639071813	0.0153599
K9JIK7	Glycophorin A	0.638569246	0.0223869
D6RIS5	Methylmalonic aciduria type A protein, mitochondrial	0.638402954	0.0134312
P04040	Catalase	0.638236898	0.00505429
B4DT46	cDNA FLJ57889, highly similar to Cytochrome b-245 light chain	0.634503665	0.00675558
E5RJR3	Methionine adenosyltransferase 2 subunit beta	0.632958065	0.0153287
G4V2I8	Anion exchange protein	0.632545568	0.0106316
Q6PCE3	Glucose 1,6-bisphosphate synthase	0.628679661	0.00683407
P02750	Leucine-rich alpha-2-glycoprotein	0.620983759	0.0417656
P07738	Bisphosphoglycerate mutase	0.620019112	0.00780953
K7WVJ5	Cytochrome c oxidase subunit 2 (Fragment)	0.620008237	0.000365803
Q8NEP7	Kelch domain-containing protein 9	0.617938076	0.034853
Q5W111	SPRY domain-containing protein 7	0.61613932	0.00902475
Q9NQT3	Alpha one globin (Fragment)	0.615531739	0.01235
P09105	Hemoglobin subunit theta-1	0.610652604	0.0138648
P00915	Carbonic anhydrase 1	0.608924235	0.00557743
B3KXV2	cDNA FLJ46110 fis, clone TESTI2033905, highly similar to Homo sapiens MYCBP associated protein (MYCBPAP), mRNA	0.605881058	0.0186889
C0IMJ3	Periostin isoform thy6	0.605482839	0.00168631
P80511	Protein S100-A12	0.605103026	0.0119478
Q86YQ1	Hemoglobin alpha-2 (Fragment)	0.601396198	0.0156982
Q9NY65	Tubulin alpha-8 chain	0.600559985	0.0368162
Q4VB86	EPB41 protein	0.600162946	0.003479
D6REQ6	Ribonuclease T2	0.593228197	0.00838654
Q86YQ4	Alpha-1 globin (Fragment)	0.589155294	0.0180772
Q9HC84	Mucin-5B	0.588815297	0.00462402
P16157	Ankyrin-1	0.588481678	0.00719774
Q8IUL9	Hemoglobin beta chain variant Hb.Sinai-Bel Air (Fragment)	0.587167978	0.00647221
E5RGQ0	Dematin (Fragment)	0.58673237	0.00406203
A0A024R674	Spectrin, beta, erythrocytic (Includes spherocytosis, clinical type I), isoform CRA_e	0.582545212	0.0237601
J3QL31	Nuclear distribution protein nudE-like 1 (Fragment)	0.581120411	0.0073303
A0A0J9YVZ3	Maltase-glucoamylase, intestinal (Fragment)	0.577986877	0.000313341
P07451	Carbonic anhydrase 3	0.573659149	0.0082248
D6CHE9	Proteinase 3	0.572504809	0.0130974
P16452	Erythrocyte membrane protein band 4.2	0.572021235	0.00640457
Q6VFQ6	Hemoglobin beta chain (Fragment)	0.559234788	0.000528424
P00918	Carbonic anhydrase 2	0.558595649	0.00530666
M0QYG6	Uncharacterized protein (Fragment)	0.552524599	0.00358503
Q4W5L2	Alpha-synuclein (Fragment)	0.549262053	0.00416355
P02549	Spectrin alpha chain, erythrocytic 1	0.546959718	0.00590205
Q4LDX3	Tyrosine-protein kinase	0.536375499	9.56722E-05
Q5XTR9	Hemoglobin delta-beta fusion protein (Fragment)	0.53538766	0.00329249
P32119	Peroxiredoxin-2	0.533829723	0.00828754
Q6IPH7	RPL14 protein	0.529004691	0.0317896
Q5BKX8	Muscle-related coiled-coil protein	0.526655148	0.00266038
B2RMN7	Spectrin, beta, erythrocytic	0.522704837	0.00569253
Q9NZD4	Alpha-hemoglobin-stabilizing protein	0.495830711	0.0123884
Q96T46	Hemoglobin alpha 2 (Fragment)	0.492646003	0.00386368
Q5T619	Zinc finger protein 648	0.492249489	0.000922952
B3VL05	Beta globin (Fragment)	0.483155107	0.00346729
Q53EU6	Glycerol-3-phosphate acyltransferase 3	0.477888471	0.00394492
B3KUX0	cDNA FLJ40831 fis, clone TRACH2012138, highly similar to Homo sapiens regulator of G-protein signalling 14 (RGS14), mRNA	0.476521157	0.00211812
Q9BXA2	Beta-globin (Fragment)	0.472636049	0.0112195
P69892	Hemoglobin subunit gamma-2	0.467543161	0.0169378
C8C504	Beta-globin	0.459856836	0.00578593
K7EP20	Dedicator of cytokinesis protein 6 (Fragment)	0.459262528	0.0211759
Q4TZM4	Hemoglobin beta chain (Fragment)	0.453442055	0.00422012
Q6EVJ6	Peptidyl arginine deiminase type IV (Fragment)	0.449186304	0.00218377
X6R5I7	Regulator of telomere elongation helicase 1 (Fragment)	0.44450906	0.0019814
H7C2R1	NADH dehydrogenase [ubiquinone] 1 alpha subcomplex subunit 3 (Fragment)	0.425402405	0.0175552
A0A024RAE0	Rh blood group, CcEe antigens, isoform CRA_b	0.412072862	0.0119529
B4E318	cDNA FLJ54246, highly similar to Homo sapiens BRCA2 and CDKN1A interacting protein (BCCIP), transcript variant C, mRNA	0.407922199	0.000239695
E9LUX2	Hemoglobin alpha-2 chain variant (Fragment)	0.391928242	0.000403159
G3V1N2	HCG1745306, isoform CRA_a	0.381102252	0.00686417
E9PJK3	Myomegalin	0.374237369	0.00223712
Q3LR79	Hemoglobin beta (Fragment)	0.373133311	0.0106368
Q52MT0	Beta globin (Fragment)	0.36879883	0.00906274
P02042	Hemoglobin subunit delta	0.340566569	0.00702286
Q8N4C8	Misshapen-like kinase 1	0.33915893	0.00302351
Q4ZGM8	Hemoglobin alpha-2 globin mutant (Fragment)	0.253926844	0.00191696
Q9UK54	Hemoglobin beta subunit variant (Fragment)	0.163325315	9.59267E-05
B2RU26	Thyroglobulin	0.163063647	0.00122084

### Bioinformatics analysis of differentially expressed proteins

**Fig. 2:**
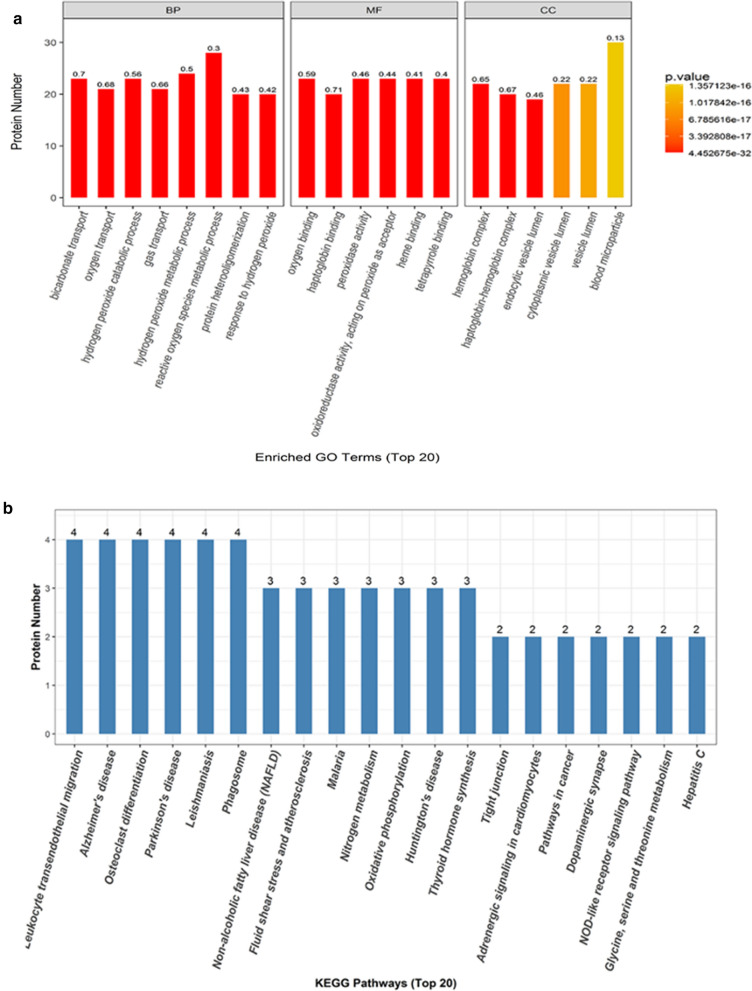
Using iTRAQ and tandem mass spectrometry determined the protein expression profiles of RPL tissue. Functional classification of differentially expressed proteins in normal and RPL human decidua, annotated using ingenuity pathway analysis: **a** Physiological System Development and Functions. **b** Disease and Disorder

**Fig. 3:**
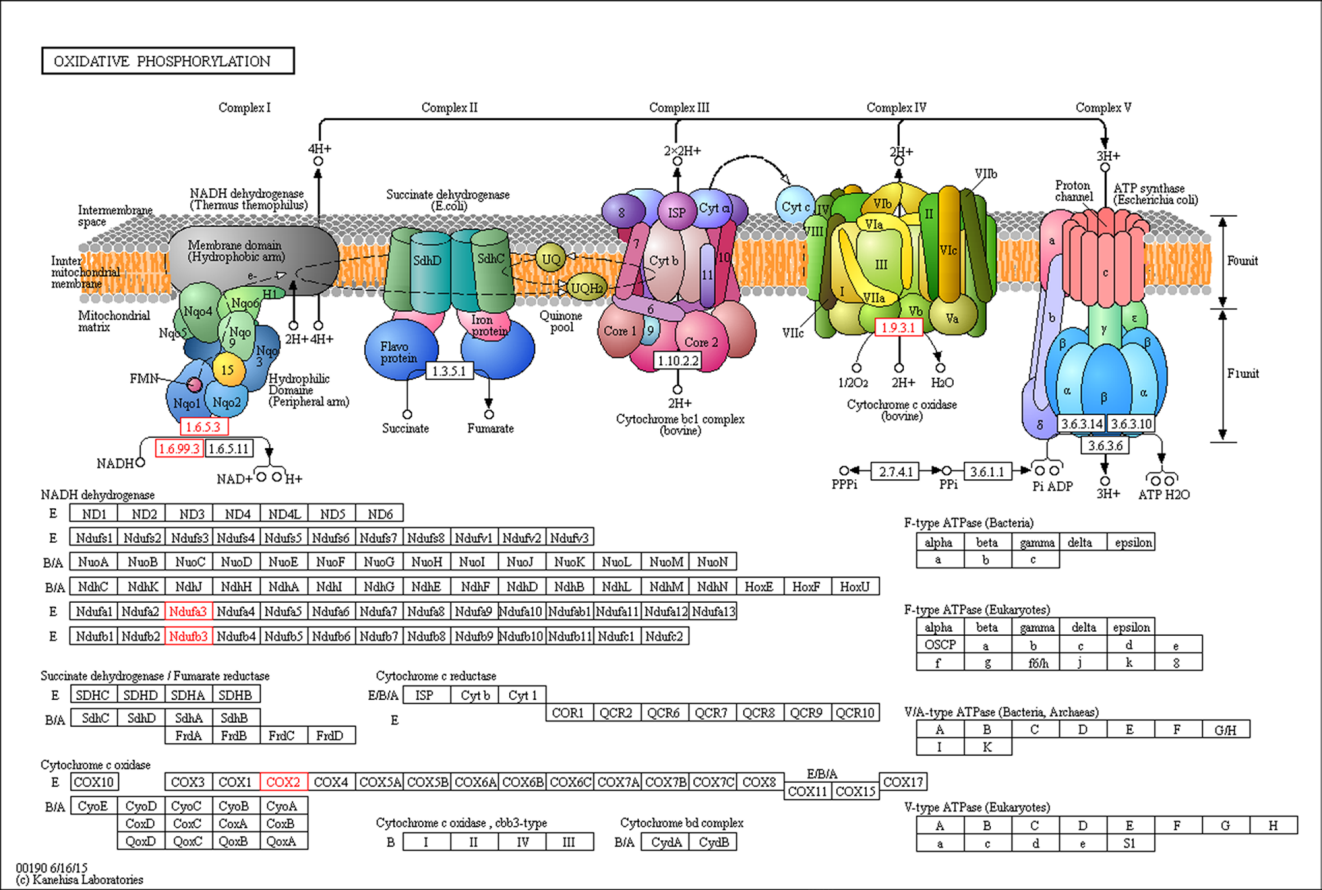
Functional enrichment analysis of differentially expressed proteins in RPL. KEGG oxidative phosphorylation pathway. Genes involved in the oxidative phosphorylation pathway are shown. Red stars denote the detected genes

Functional enrichment analyses, including GO enrichment and KEGG pathway enrichment, were performed on the resulting gene signature. GO analysis was performed in three categories, including biological processes (BP), molecular functions (MF), and cellular components (CC). Based on their associated p values, we identified the top 20 subcategories of “Physiological System Development and Functions” (Fig. 2a) and the top 20 subcategories of “Disease and Disorder” (Fig. 2b) associated with these differentially expressed proteins. This analysis indicated that some of the differentially expressed proteins were related to oxidative phosphorylation, which including NDUFB3 and COX-2 (a subcategory of “Disease and Disorder”). KEGG pathway enrichment analysis demonstrated that of the differentially expressed genes are involved in the oxidative phosphorylation pathway (Fig. 3).

### NDUFB3 is up-regulated in decidual cells from RPL patients

**Fig. 4:**
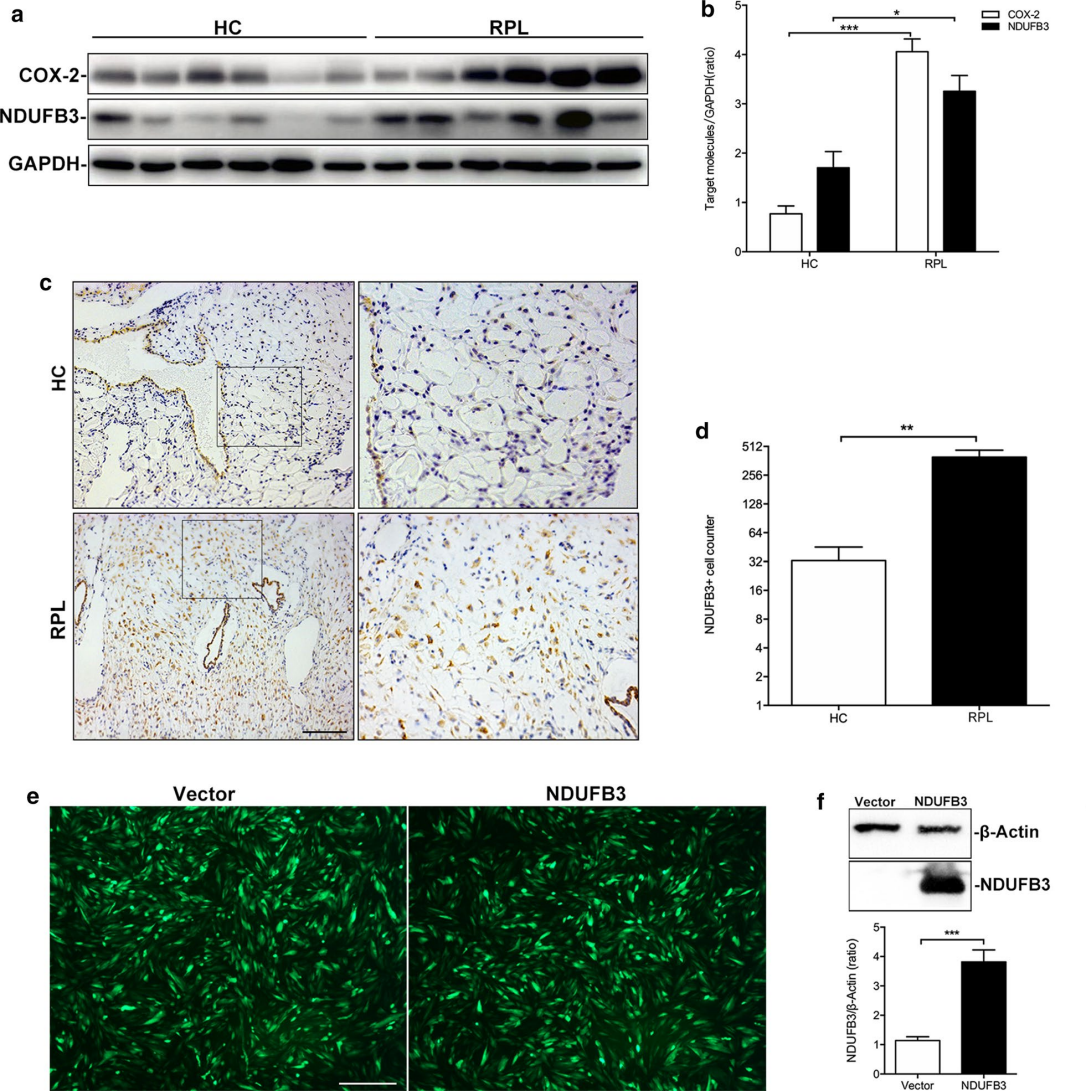
NDUFB3 and COX-2 were up-regulated in decidua in patients with RPL. Validation of the differential expression of NDUFB3 and COX-2 proteins by Western blotting, immunohistochemistry. **a**, **b** NDUFB3 and COX-2 expression levels were significantly increased in RPL group. **c**, **d** NDUFB3 expression was significantly increased in RPL group (bar = 200um, HC: healthy pregnant women control). **e** Infection efficiency in NDUFB3 adenovirus infected primary DSCs (bar = 50um). **f** Western blotting analysis of NDUFB3 expression in DSCs infected with NDUFB3-overexpressing adenovirus after 48 h (***p < 0.001 compared to vector group)

We performed Western blotting analyses to verify key proteomic differences discovered by TMT analysis. We mainly analyzed differentially expressed proteins associated with the process of oxidative phosphorylation. First, we confirmed that NDUFB3 and COX-2 were significantly altered post-RPL. Expression of these proteins could affect mitochondrial respiratory chain function. As shown in Fig. 4, NDUFB3 and COX-2 expression levels were significantly increased in decidua tissue from the RPL group (Fig. 4a, b). NDUFB3 expression was also increased in decidua from RPL patients (Fig. 4c, d). As NDUFB3 was expressed in the decidua, we next investigated whether NDUFB3 is involved in the cellular activity, oxidative stress capacity of decidua. To this end, we used DSCs were infected with NDUFB3-overexpressing adenovirus or the vector. The infection efficiency of NDUFB3-overexpressed adenovirus was verified (Fig. 4f).

### NDUFB3 inhibits oxidative stress and reduces the cell vitality of DSCs in vitro

**Fig. 5:**
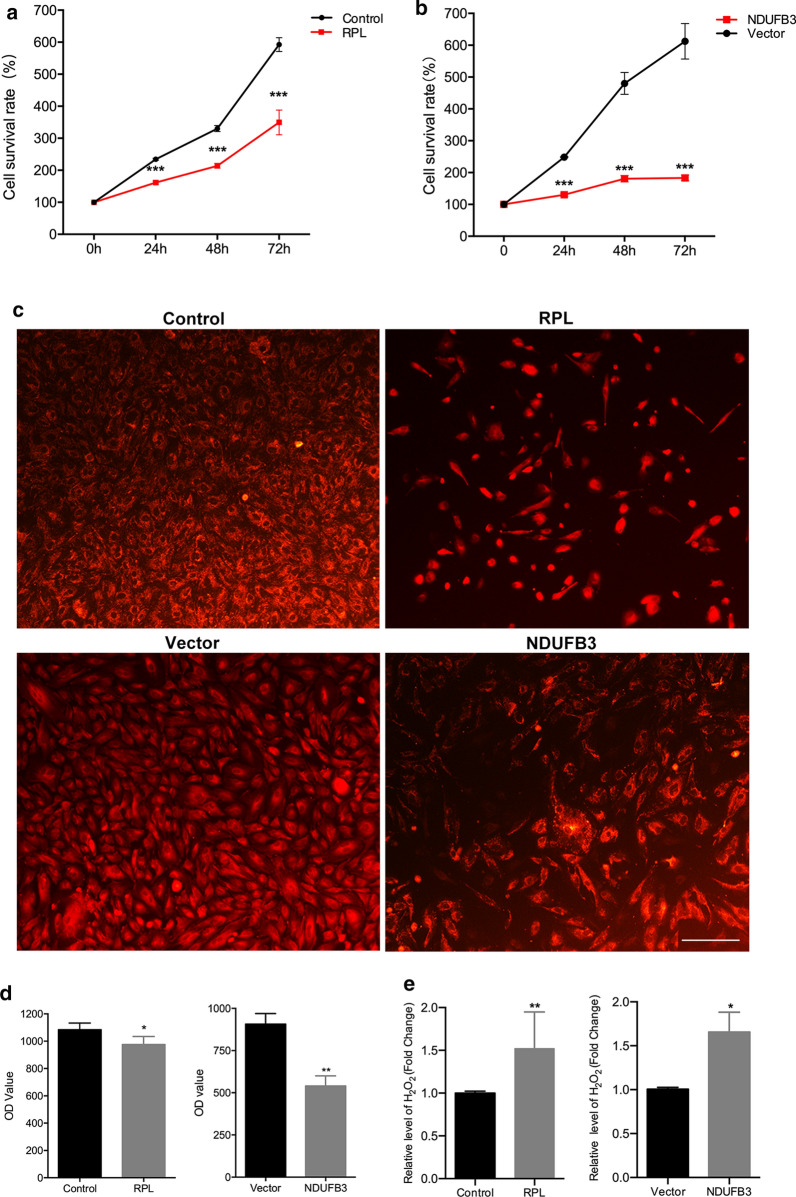
NDUFB3 inhibits the function of decidua in vitro.** a**, **b** CCK-8 assay of NDUFB3-overexpressing of DSCs and RPL group from patients and their respective control groups. **c** Mitochondrial membrane potential was detected by mito-tracker Red CMXRos assays. Immunofluorescence staining analyses of DSCs revealed expression of NDUFB3 (red). Original magnification: × 100. Scale bar = 100 μm. **d** The OD value analysis of NDUFB3 expression. **e** Levels of H_2_O_2_ in DSCs after treatment 48 h. Data are presented as the mean ± SD. *p < 0.05, **p < 0.01, ***p < 0.001 compared to control or vector group

As NDUFB3 was expressed in the decidua, we next investigated whether NDUFB3 is involved in the cell vitality and oxidative stress capacity of DSCs. The CCK8 assay demonstrated that overexpression of NDUFB3 significantly decreased the cell vitality of DSCs compared to RPL (Fig. 5a, b). To further examine the effects of NDUFB3 and COX-2 on mitochondrial function**,** mito-tracker Red CMXRos assays were conducted. The results revealed that NDUFB3 overexpression significantly decreased the mitochondrial membrane potential. So that the mitochondria can't maintain their normal function (Fig. 5c, d). In addition, we further examined the oxidative stress ability of DSCs after overexpression of NDUFB3. Hydrogen peroxide is a by-product of reactive oxygen metabolism and is a key regulator in many oxidative stress responses. Hydrogen peroxide is also closely related to cell apoptosis and cell proliferation. The results revealed that NDUFB3-overexpression significantly increased the level of H_2_O_2_ of DSCs (Fig. 5e). These results suggested that NDUFB3 might play a key role in decidua function.

## Discussion

Human reproduction is relatively inefficient [24]. Miscarriage, the pathogenesis of which remains unclear, is a common and difficult complication of human pathological reproduction. Many factors are thought to be involved in the pathogenesis of recurrent pregnancy loss and accumulating evidence suggests that oxidative stress (OS) is a common pathophysiological feature in the various etiologies of early pregnancy loss [25–28]. When human and animal cells are stimulated with nitric oxide, calcium, pathogens or other internal or external factors, the balance between the oxidant and antioxidant systems is destroyed, thereby promoting the production and accumulation of intracellular reactive oxygen species (ROS), and eventually leading to oxidative stress buildup in the body [29, 30]. Although trophoblast cell damage has been attributed to multiple biochemical pathways and mechanisms of action, all of these mechanisms involve the formation of ROS. Nicotinamide adenine dinucleotide phosphate (NADPH) is a major source of both physiologic and pathophysiologic ROS generation. ROS can induce dysfunction and damage of trophoblast cells at the maternal–fetal interface [31].

Our study determined that ndufb3 is an important gene involved in regulating RPL risk, as it is involved in both the mitochondrial function and oxidative stress pathways. Specifically, ndufb3 in combination with cox-2 regulates mitochondrial oxidative stress and stabilizes mitochondrial membrane potential so that decidual cells can grow normally. Interestingly, compared with many other cell types, trophoblast cells may be at a higher risk of oxidative stress and may have an increased sensitivity to apoptosis. It is now widely accepted that oxidative stress is one of the most important mechanisms contributing to mitochondrial damage.

Oxidative stress can lead to DNA strand breakages, locus mutations, double-strand aberrations and damage in the form of proto-oncogene and tumor suppressor gene mutations. DNA is also subjected to X-rays, ultraviolet radiation, intercalating agents and other physical and chemical factors, all of which are known to cause DNA damage in vitro. DNA damage can also result in oxidative stress production. The loss of antioxidant defenses has been shown to be associated with recurrent pregnancy loss. Oxidant/antioxidant imbalances have also been proposed to be associated with pregnancy loss [32]. A previous study demonstrated that sustained endoplasmic reticulum stress induces apoptosis via overexpression of Caspase-4 and Caspase-12 in early pregnancy loss decidua [33]. More recently, another study found that peroxiredoxin 2 downregulation plays a role in recurrent miscarriage through regulation of trophoblast proliferation and apoptosis via effects on ROS metabolism [34].

Mitochondria play a central role in mammalian cell energy metabolism, which is involved in both the life and death of cells. Mitochondria ensure a proper balance between pro- and anti-apoptotic factors and generate ATP through the oxidative phosphorylation (OXPHOS) system. Therefore, mitochondria are sensitive to a variety of signals that are critical in regulating their functionality [35, 36]. The regulation of mitochondrial function is an intricate process complicated by the participation of many resident and nonresident mitochondrial proteins.

The deterioration of mitochondrial stress response pathways leads to defects in mitochondrial function, which has become a prominent signature of metabolic, cardiovascular, renal, inflammatory, reproductive, muscular, and neurodegenerative diseases as well as infections and cancers [37, 38]. Our work addresses several of the most prominent proteins involved in regulating mitochondrial function and provides insight into their biological mechanisms. Firstly, our study shows that the overexpression of elevated NDUFB3 expression may be a potential cause of recurrent pregnancy loss. Second, according to KEGG pathway enrichment analysis, the ndufb3 and cox-2 genes are involved in the oxidative phosphorylation pathway. Finally, we analyzed the association of these proteins with oxidative phosphorylation and found that NDUFB3 is differentially overexpressed in decidua. We also detected mitochondrial membrane potential by JC-1 and found that mitochondrial membrane potential significantly increases following overexpression of ndufb3; this would further induce oxidative stress and apoptosis in decidual cells. ndufb3 dysfunction may be an important risk factor of various problems involved in RPL including mtDNA-induced mitochondrial damage and cell death and their associated repair pathways. Our findings provide a deeper view of these processes and further illuminate their role in the underlying pathogenesis of RPL.

Nevertheless, this study was limited in several aspects. First, we only considered maternal contributions to RPL, as the microenvironment within the woman’s body is critical for determining fetal viability. Since NADH polymorphisms play an important role in determining the gestational status of women, we determined the genetic effects of ndufb3 and cox-2 alleles in order to estimate the female contribution to RPL risk among a Chinese population. However, the contribution of the male karyotype to increased RPL risk cannot be ignored, and later studies could potentially address this limitation. Additionally, our study only examined the contributions of ndufb3; additional genes should certainly be taken into consideration in future studies. Last, environmental factors should also be considered, as unfavorable conditions could facilitate the occurrence of RPL.

## Conclusions

In the present study, we demonstrated that NDUFB3 is highly expressed in decidual tissue of RPL patients. Interestingly, notably higher levels of NDUFB3 were produced by decidual tissue in the RPL group compared with the con group. In addition, overexpression of NDUFB3 using an adenovirus effectively inhibited DSCs outgrowth, mitochondrial membrane potential and oxidative stress function in vitro. The correlation between the increased expression of NDUFB3 and RPL provides a pathological criterion that may be applied for the diagnosis and treatment of potential miscarriages.

## Data Availability

Please contact author for data requests.
